# A composite hydrogel membrane with shape and water retention for corneal tissue engineering

**DOI:** 10.1016/j.heliyon.2023.e17950

**Published:** 2023-07-05

**Authors:** Li Jiang, Xiaoli Dong, Luxia Chen, Ruifang Han, Pen Hao, Liming Wang, Juan Gao, Xi Chen, Xuan Li

**Affiliations:** a Clinical Collage of Ophthalmology, Tianjin Medical University, Tianjin, China; b Tianjin Key Laboratory of Ophthalmology and Visual Science, Tianjin Eye Institute, Tianjin Eye Hospital, Tianjin, China; c Nankai University Affiliated Eye Hospital, Tianjin, China

**Keywords:** Cornea transplantation, Sodium alginate, Spherification, Hydrogel, Tissue engineering

## Abstract

Tissue engineering (TE) cornea is one of the most potential alternatives to the shortage of corneal donors in cornea transplantation. Sodium alginate (SA) hydrogel is commonly used as scaffold in TE. Herein, we present an approach to construct a composite hydrogel, which with SA fiber skeleton structure for shape retention and gelatin surface modification for water retention. The light transmittance, water retention rate, and swelling rate of hydrogels were characterized, and the tensile mechanical properties were also investigated. Keratinocytes were treated with material extract liquor and the results showed that the gelatin modified SA hydrogel has good cytocompatibility. Furthermore, human corneal stromal fibroblasts (HCSFs) from the lenticules were implanted on the surface of gels, and the SA-gelatin hydrogel significantly improved the adhesion and spreading of HCSFs. Finally, we discussed the improvement and application prospect of the composite hydrogel as cornea equivalents.

## Highlights

• The hydrogel could be fabricated by natural materials and mature cross-linking methods.
• The hydrogel showed the property with shape retention under rapid cross-linking condition.
• The hydrogel showed good corneal cell compatibility and cell-friendly interface.

## Graphical abstract

**Figure:**
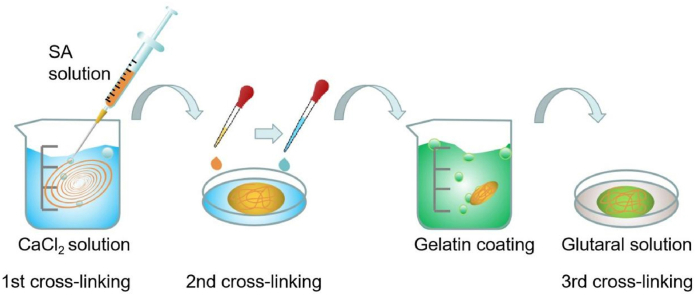

## 1 Introduction

Corneal blindness is one of the major causes of global vision impairment. According to the World's First World Vision Report by World Health Organization in 2019, approximately 2.2 billion people are suffering from visual impairment, mostly caused by corneal-related diseases, which results in 60 million corneal blindness patients in the world [1]. Cornea transplantation is the main therapeutic option for severe corneal damage caused by traumatic infection and chemical injury [2]. However, as the gold standard, high-quality donated corneas are still very limited compared to the number of patients who need transplantation [3]. So the appropriate cornea equivalents are urgently needed to meet the corneal microenvironment adaptability and reduce the waiting times of patients.

Alginic acid is a natural linear polysaccharide extracted from *Marine brown algae*, the water solution of its salt derivatives (sodium alginate, SA) could be cross-linked with divalent cation (e.g. Ca^2+^ and Mg^2+^) to form a three-dimensional network structure——SA-hydrogel. Due to its good biocompatibility, cost-effective, non-toxic, and biodegradable properties, SA-hydrogel has great potential in scaffolds preparation, including joints, cartilage, neural, ear, heart, cornea [4], and cell delivery vehicles [5]. More importantly, the proper light transmission of SA-hydrogel make it become one of the most suitable materials for cornea scaffold [6].

The simplest method of SA gelatinization is dropping a certain concentration of SA solution into the cationic solution, then the droplets could form liquid-core hydrogel beads in the rapid cross-linking process, this phenomenon is also known as spherification in molecular gastronomy [7]. Spherification means the liquid is reshaped into a sphere along with the process of surface gelation. The gelation occurs first on the surface, then to the inside of the droplets. Take advantage of this phenomenon, the beads could even encapsulate drugs, liquid foods/juice or bioactive substances [8].

However, spherification would also make an effect on SA-hydrogel membrane preparation. The shape of the SA-hydrogel membrane made by the traditional crosslinking method (mixing SA and ion solution in a mold) tends to be a sphere (especially on the edge of the mold), which makes it difficult to maintain the thickness uniform of the membrane. When the volume of the alginate solution gets smaller, the sphere trend is more obvious. It makes it difficult to precisely fabricate the tiny and thin scaffolds like corneal tissue. Moreover, the formed hydrogel beads or membranes could contain inadequately crosslinked solutions.

Tissue engineering provides new ideas for the preparation of corneal substitutes. Fiber-reinforced strategy, including electrospinning technique, is commonly used by many researchers in bionic construction [9,10]. Recently, a fiber-reinforced gelatin methacrylate (GelMA) hydrogel [11]was fabricated by a direct writing device and cross-linked under 365 nm light. The addition of fibrous grid poly (ε-caprolactone)-poly (ethylene glycol) reinforced the strength of the hydrogel scaffold and have a synergistic effect with chemical factors to induce the regeneration as the results of intrastromal keratoplasty in rats.

Inspired by this, we tried to make a fibrous structure inside of hydrogel to reduce the effect of spherification on the shape of SA-hydrogel, then modified the surface with gelatin for water retention. Herein, we prepared an SA-gelatin composite hydrogel with a fiber skeleton structure by diverse cross-linking methods.

The preparation process of fibrous skeleton composite hydrogel.

## 2 Materials and methods

SA & gelatin powder (Shanghai yuanye Bio-Technology, Shanghai, China), 2.5% glutaraldehyde solution (Solarbio, Beijing, China), CaCl_2_ (Sangon Biotech, Shanghai, China), corneal trephines (φ10 mm, Xiehe, Suzhou, China).

### 2.1 Cell culture and characterization

#### 2.1.1 Keratinocytes (KCs) culture

KCs were obtained from ATCC and the culture medium was RPMI1640 (HyClone, USA) supplemented with 10% fetal bovine serum (FBS, Gibco, USA) and 1% pennicillin & streptomycin (PS, Gibco, USA).

#### 2.1.2 Human corneal stromal fibroblasts (HCSFs) culture and characterization

The human corneal lenticules were collected from 3 patients with myopia in small incision lenticule extraction (SMILE) refractive surgery (age range: 19–28 years, optical zone of lenticules: 6.6 mm, thickness: Min 10 μm, Max 140 μm) at Tianjin Eye Hospital. This study was approved by the Medical Ethics Committees of Tianjin Eye Hospital (Study number: KY-2023036) and was conducted according to the declaration of Helsinki's tenets. HCSFs were obtained by tissue cultivation. Briefly, lenticules were transferred to the sterile tubes and transported to the cell laboratory within 2 h on the ice, then cut the tissues into pieces and spread them on the bottom of the T25 flasks. After the tissue pieces were firmly attached, DMEM/F12 medium (HyClone, USA) with 10% FBS was added. After 11 days, cells were harvested and passaged in time.

Immunofluorescent staining was used to identify HCSFs. Firstly, cells were planted on the glass bottom cell culture dish (φ 15 mm, Nest, Wuxi, China) overnight, then were washed with phosphate-buffered saline (PBS) three times, 4% tissue fixation fluid (Biotopped, Beijing, China) was added, then cells were transparent with 0.1% Triton X-100 and incubated with 5% BSA to block the non-specific sites, then incubated with *anti*-Vimentin antibody (1:500 dilution, Abcam, Cambridge, MA, USA) at 4 °C overnight. Washed with PBS three times and incubated with AlexaFluor 488-conjugated rabbit anti-mouse IgG (1:1000 dilution, Invitrogen, USA) for 1 h at RT, then washed with PBS three times, and counterstained with DAPI for 10 min and examined under the confocal microscopy (Leica TCS SP8).

### 2.2 Preparation and characterization

#### 2.2.1 Preparation of composite SA-hydrogels

1% (w/v) SA, 10% gelatin, 1% CaCl_2_, and 0.1% glutaraldehyde solutions were prepared in ultrapure water respectively. Then SA solution was heated to 50 °C in a water bath and loaded into a 2.5 mL medical syringe connected with a lacrimal duct needle (φ 0.5 mm).

Fibrous gels were fabricated according to the wet spinning principle [12]. Briefly, SA solution was injected into CaCl_2_ solution quickly to form fibrous gels. The fibrous gels were washed with ultrapure water and infused with extra SA solution in a 48-well plate to prepare circular gels, then cross-linked in CaCl_2_ solution again for 1 h. The gels were dipped into gelatin solution, then crosslinked by glutaraldehyde solution for 1 h, immersed into PBS solution overnight, and washed with PBS three times.

The fiber structure hydrogel with gelatin coating was denoted as AlgF-gel and fiber structure hydrogel was denoted as AlgF in experiments.

#### 2.2.2 Effect of water content on hydrogels transparency

The transparency of hydrogels was examined by measuring the light transmittance. Cylindrical gels with a diameter of 10 mm were prepared by trephine and were placed in a 48-well plate to study.

The absorbance A was measured at 405, 450, 490, 570, and 630 nm with a microplate analyzer (BioTek Instruments, USA). The transmittance (T) values were calculated by formula T (%) = 1/10^A^ × 100.

In order to evaluate the effect of water content on transparency, the hydrogels (*n* = 3) were treated with glycerol before optical evaluations according to the literature [13], then tested absorbance at 490 nm in different time points (0.5, 1, 1.5, 2.5, 4.5 h).

#### 2.2.3 Swelling rate and water retention rate

To test the water absorption of the hydrogel, the swollen gels (φ10 mm) were incubated in a 37 °C incubator (RH 29%–69%), and the weight of each gel at different time points was recorded, the xerogels were also weighed after incubating in 50 °C oven for 3 h.

The swelling rate formula is S (%) = (W–W_0_)/W_0_ × 100 (S means the swelling rate, W means the mass of hydrogel after swelling equilibrium, W_0_ means the mass of xerogel).

And water retention is calculated as W (%) = (W_x_ − W′)/(W − W′) × 100 (W_x_ means the mass of hydrogel after x h treated, W means the mass of wet hydrogel, W’ means the mass of xerogel).

#### 2.2.4 Mechanical testing

For mechanical testing, specimens (φ10 mm, *n* = 8) were prepared and the thickness was measured by optical coherence tomography (OCT, YG-100K pro, TowardPi, Beijing) images, mechanical properties were determined by a tensile test machine (BioTester, CellScale, Waterloo, Canada) at an elongation speed of 75 μm/s in a 37 °C saline bath. The maximum tensile strength, elongation at break, and elasticity modulus were recorded and calculated.

#### 2.2.5 Cell viability and adhesion tests

Cell viability was determined using CCK-8 reagent. Gels were sterilized by UV irradiation before cell culture, then HCSFs (passage 4, P4) were seeded on each gel at 2 × 10^3^ cells in a 96-well plate and were cultured with material extract (1.5 cm^2^/mL, 37 °C, 24 h) for 24 h, CCK-8 reagent (Takara Biomedical Technology, Beijing, China) was added directly into the wells and incubated for 2 h in cell incubator, the light absorption value at 450 nm was detected.

HCSFs (P5) were seeded on the gels, after 24 h incubation at 4 × 10^4^ cells in a 48-well plate, cells were fluorescently stained with a live & dead staining kit (Proteintech, Rosemont, IL, USA) and photographed. The culture medium was DMEM/F12 supplemented with 10% FBS and 1% PS.

### 2.3 Statistical analysis

All data were expressed as mean ± standard deviation (SD). Statistical analysis was performed using Student *t*-test (GraphPad Prism, version 7.00, La Jolla, CA). Data are shown as mean ± standard deviation (SD) for quantitative variables. A value of *P* < 0.05 was considered statistically significant; n.s. indicates not statistically significant (*P* > 0.05).

## 3 Results and discussion

### 3.1 Isolation and characterization of HCSFs

SMILE is one of the new keratorefractive techniques, and the surgical procedure of SMILE is widely adopted in myopia correction in the last decade. Derived stromal lenticules during surgery provide very suitable material for corneal stromal equivalent studies, and clinical application [[14], [15], [16], [17]], especially for human primary keratocyte culture [18]. In the normal cornea, keratocytes are in a state of quiet and arrested in the G0 phase, when cultured *in vitro*, keratocytes can be activated and easily transform into fibroblast-like cells under the condition of serum media, which could promote cell division [19]. Cells isolated from lenticules were cultured in DMEM/F12 medium containing 10% FBS and 1% PS. To identify the types of primary cultured cells (P5), the expression of Vimentin, specifically expressed in fibroblasts, was investigated by immunofluorescence staining.

**Fig. 1:**
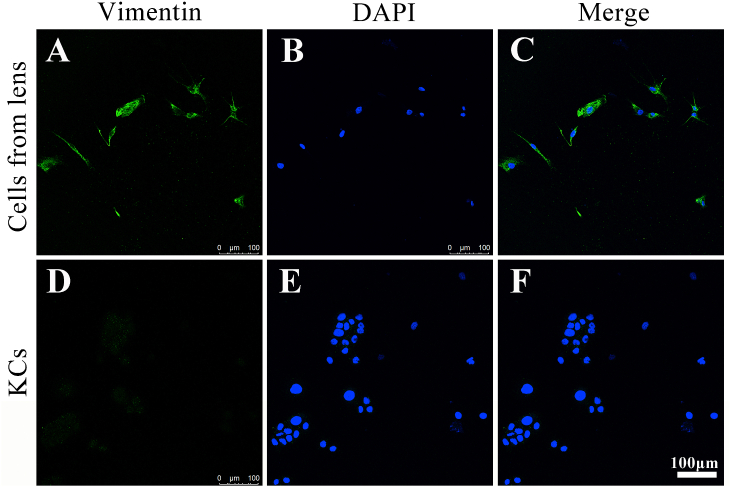
Immunofluorescence staining of cells cultured from lens (A–C) and KCs (D–F). Vimentin (green), DAPI (blue), the scale bars are 100 μm. (For interpretation of the references to color in this figure legend, the reader is referred to the Web version of this article.)

As shown in Fig. 1, the marker of HCSFs -Vimentin, was strongly expressed in the cultured cells (Fig. 1A–C) compared with KCs as control (Fig. 1D–F), indicating that they were corneal stromal cells.

### 3.2 Characterization of hydrogels

In order to enhance SA solution liquidity, we used a water bath to make the SA solution maintain 50 °C before hydrogel preparation.

**Fig. 2:**
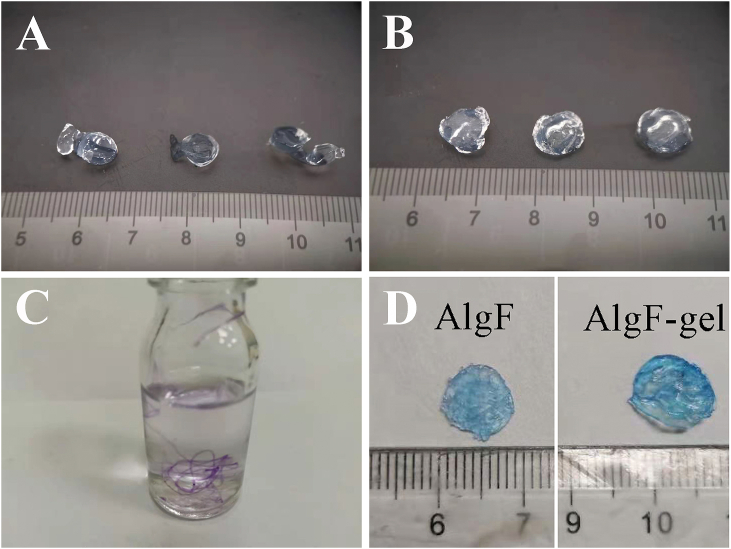
The macroscopic shape of (A) SA and (B) AlgF gels, (C) The macroscopic morphology of SA fibrous gel (stained purple for visualization purposes only), (D) The gels stained with Coomassie blue. (For interpretation of the references to color in this figure legend, the reader is referred to the Web version of this article.)

The results of Fig. 2A&B showed that the swelling internal fibers contribute to reducing the effect of spherification during gel formation in a 48-well plate, and the fibrous hydrogel could maintain relatively good shape retention properties. The effect was also obvious during the preparation in a dish (φ 9 cm).

Corneal trephine is a common surgical instrument in ophthalmic surgery, which is suitable for preparing circular tissues with different diameters for transplantation. The circular hydrogels (φ 10 mm) used in follow experiments were prepared in a dish (φ 9 cm) and cut with a corneal trephine (φ 10 mm).

To clearly show the macroscopic morphology of cross-linked SA fibrous gel, the SA solution was pre-mixed with Coomassie blue before being loaded into a syringe. Fig. 2C showed the extruded SA solution was capable of rapid gelation and form a continuous fibrous morphology in CaCl_2_ solution.

As a commonly used material for scaffold preparation in tissue engineering, the dipping coating is a simplified and efficient measurement in surface modification methods. For example, multiple layers of hybrid hydrogels can be formed by repeated dipping operations [20]. By Coomassie blue staining, AlgF-gel membrane showed a darker blue color compared with AlgF membrane in Fig. 2D, indicating that the gelatin was coated on the surface of Alg gel successfully, by dipping the gels into crosslinking agent (0.1% glutaraldehyde solutions), and created a thin layer on the surface of gels.

**Fig. 3:**
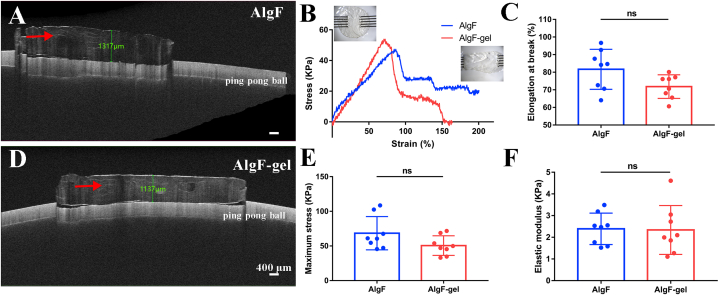
(A, D) OCT images of hydrogels, (B) Stress and strain curves (C) Elongation at break, (E) Maximum stress, (F) Elastic modulus of gels (red arrows showed the fiber structure in hydrogels). (For interpretation of the references to color in this figure legend, the reader is referred to the Web version of this article.)

The cross-section morphology of the membranes was further observed by OCT instrument (Fig. 3A&D). OCT imaging is characterized by non-contact and non-destructive, and with higher detection sensitivity and resolution than common measurement methods. Herein, OCT images of hydrogels were also used to measure thickness, characterized the distribution of fibers in gels and assess internal integrity. In the test, the gels were attached to a ping pong ball, which was used to simulate eyeball. The results of OCT images showed that the thickness of fibrous hydrogel could reach 859 ± 288 μm for AlgF and 1096 ± 96 μm for AlgF-gel, which were similar to the peripheral part of native cornea (1000 μm).

The tensile strength was measured by a tensile test machine to investigate the effects of fiber structure on the gel's mechanical properties. The tensile stress and strain curves were shown in Fig. 3B. The elongation at break of AlgF group was 81.65 ± 11.40%, maximum stress was 68.45 ± 24.04 KPa, elastic modulus was 2.39 ± 0.73 KPa; and elongation at break of AlgF-gel group was 71.81 ± 6.70%, maximum stress was 50.56 ± 14.07 KPa, the elastic modulus was 2.34 ± 1.13 KPa, (Fig. 3C, E, F). The insert pictures in Fig. 3B showed the status of gels before and after the test.

There was no significant difference between the two groups. It means that the fiber structure in gels did not affect the tensile strength of hydrogel. But compared with the parameter of native cornea (tensile strength: 3–5 MPa [21], tensile modulus: 121.8 KPa [22]), the mechanical strength of hydrogels is still far from clinical application.

Cornea is the outermost layer of human eyes and the main refractive mediator that focuses light on the retina of the eyes [23]. Injury, inflammation, malnutrition, tumors, and other diseases could damage corneal cells and disrupt stroma function, leading to detrimental effects on vision [24].

**Fig. 4:**
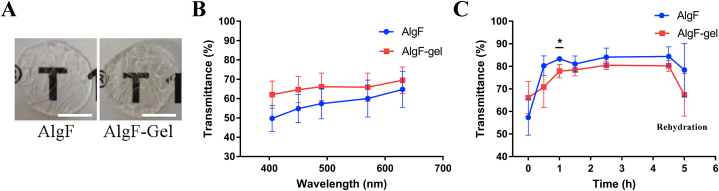
(A) Digital images of hydrogels, bar = 5 mm, (B) Light transmittance of gels, (C) Light transmittance of gels in different degrees of dehydration and rehydration at 490 nm, *P < 0.05.

SA is a common food additive, and the natural transparency after crosslinking also made SA gel tremendous potential in corneal equivalent preparation. The AlgF and AlgF-Gel gels (Fig. 4A) were also prepared to assess the transparency property, the transparency of the AlgF-Gel group (62.05%–69.46% at λ from 405 to 630 nm) was higher than that of the AlgF group (49.73%–64.76% at λ from 405 to 630 nm) (Fig. 4B). A certain amount of water in the cornea (about 75%) is important to maintain its transparency, so we supposed that the water holding in AlgF-Gel was suitable for light transmittance improvement, although the exact range and distribution of water content is unclear at present.

The water absorption of glycerol could reduce the gel's non-specific swelling by reducing the water content. The relationship between water retention and transmittance was also investigated by glycerol treatment (Fig. 4C). When immersed in glycerol over 0.5 h, the transmittance of AlgF group at 490 nm increased to 80.25%, and the transmittance of AlgF-Gel group reached 80.55% after treated 2.5 h, close to the visible spectrum of the natural cornea (87%) [25]. Interestingly, when membranes were immersed in dH_2_O to rehydrate, the transmittance would reduce, indicating the water content have a huge impact on gel transparency.

**Fig. 5:**
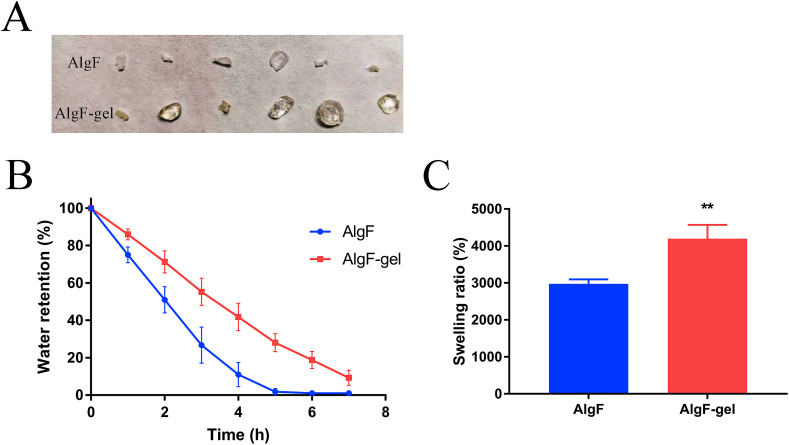
(A) Diaphragm shape after water loss for 6 hs, (B) Water retention rate, *n* = 4, (C) Swelling rate of gels, *n* = 4, **P < 0.01.

As one of the important characteristics of hydrogel, water retention capacity is closely related to the maintenance of cell proliferation and tissue regeneration. Therefore, the swelling rate of gels and water retention were also examined, as shown in Fig. 5C, the swelling ratio of AlgF-Gel (4198 ± 376%) was significantly higher than AlgF group (2979 ± 120%) and native cornea (1450 ± 59%) [11], illustrating the cross-linked gelatin coating could maintain moisture on the gel surface. And water retention rate of AlgF group decreased faster with prolonged time (Fig. 5B).

Gelatin is derived from partial hydrolysis of collagen, side chains of gelatin contain a variety of hydrophilic groups, such as amino group, carboxyl group, etc, which contribute to the affinity between gelatin and water molecules and reduce water loss of hydrogel in a relatively dry environment. As shown in Fig. 5A, after 6 h treatment in the incubator, AlgF membrane lost most water and the gels were visibly shrunken, and AlgF-Gel showed a higher humidity tolerance compared with AlgF groups.

The lack of tear moisture on the ocular surface would affect tissue repair after transplantation. The coating of gelatin increased the water retention rate and swelling rate of the gels significantly. The higher swelling ratio of AlgF-Gel means it could hold more water, and longer water retention time contributed to resisting the dryness of the external environment, and accelerate the healing process of ocular surface wounds.

#### 3.2.1 Cell compatibility and adhesion on gels

SA gels are bio-inert and the surface of SA hydrogel lacks cell adhesion sites, which is not suitable for cell adhere and growth. As uncrosslinked gelatin is instability on long-term incubation and easily degraded by enzymes *in vivo*. We used the chemical crosslinking method to stabilize the gelatin form.

**Fig. 6:**
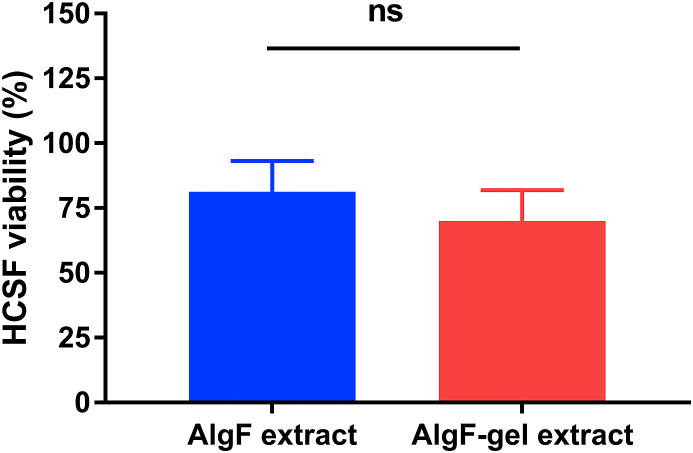
Cellular compatibility of gel material extracts.

Glutaraldehyde is the most commonly used chemical cross-linking agent for improving the mechanical properties of collagen-based materials, due to its ready availability and high efficiency [26], but it shows adverse effects on cells at higher concentrations in past studies [27]. Herein, cell compatibility of gels was assessed by using gel extracts culture. High viability of HCSFs was observed in AlgF-gel group, as shown in Fig. 6. This indicated the residual amount of glutaraldehyde in the gel would not affect cell viability *in vitro* culture.

In corneal TE, corneal stromal cells are commonly used as seed cells [28]. For example, Isaacson et al. [6]prepared a 3D-printed cornea equivalent with SA-collagen bioink and embedded with corneal stromal cells. The 7-day survival rate of the cells in composite hydrogel after bioprinting reached 83%. Here we chose HCSFs as seed cells in the cell adhesion test.

**Fig. 7:**
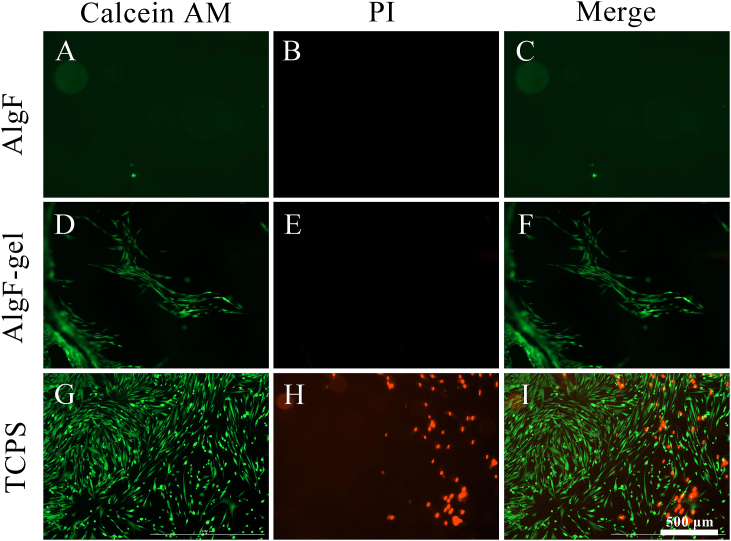
The viability and adhesion of HCSFs on gels assessed by live & dead staining assay (A–C: AlgF, D–F: AlgF-gel, G–I: TCPS, Green - calcein: living cells, Red - propidium iodide: dead cells, scale bars: 500 μm). (For interpretation of the references to color in this figure legend, the reader is referred to the Web version of this article.)

In Fig. 7, immunofluorescence images showed the cell state and morphology of HCSFs on gels after 24 h of incubation, green fluorescence indicated the living cells and red fluorescence showed the dead cells. Compared with AlgF group (Fig. 7A–C), the cells distribution was only observed in AlgF-Gel group (Fig. 7D–F), and the morphology of the HCSFs spread to be spindle-shaped, which was similar to the appearance of control (Fig. 7G–I).

Constructing composite scaffolds with bioactive components is a common strategy to improve implant cytocompatibility, which lack of biological activity [29]. Alginate is known to be biologically inert and has low cellular adhesion, which is a major disadvantage to cellular proliferation and differentiation [30].

As far as we know, cell adhesion and growth on material surfaces is one of the most important objectives of tissue engineering. Gelatin is the hydrolyzed derivative of collagen (the major component of cornea matrix), also used to prepare the scaffolds. Furthermore, functionalized gelatin, such as GelMA is also commonly used as scaffold to repair corneal tissue [[31], [32], [33], [34], [35]]. Besides, as a biocompatibility, biodegradable, and inexpensive biomaterial, gelatin coating is also widely used to promote cell adhesion in Petri dishes.

Herein, the results indicated that HCSFs were very difficult to grow on the alginate gels, while the gelatin surface coating would improve the bioactivity and contribute to the adhesion of cells significantly.

## 4 Conclusions

As a readily available material, the fast gelatinizing ability at room temperature (RT) of SA provides it pliability and gelling adeptness for extrusion additive manufacturing/bioprinting [36,37]. In this study, we demonstrated the feasibility to fabricate an alginate gel as a corneal equivalent with a fibrous skeleton structure by rapid gelation via cross-linking under mild conditions. The composite hydrogels showed the property with shape and water retention and *in vitro* tests also demonstrated good cell compatibility and cell-friendly interface.

At present, there is still no engineered cornea or equivalent available for clinical application [34], the poor mechanical properties are also one of the reasons. We would further optimize the preparation process of gels (reduce fiber caliber and increase density in hydrogel) to improve its mechanical properties and light transmittance, explore the *in vitro* degradation characteristics of hydrogels and the rabbit corneal pocket model would be used for test biocompatibility *in vivo* in the next step experiment. It would be a promising strategy and there is great potential in the preparation of TE corneal in the future.

## Financial disclosure

No author has a financial or proprietary interest in any material or method mentioned.

## Author contribution statement

Li Jiang, Xiaoli Dong: Conceived and designed the experiments; Performed the experiments; Analyzed and interpreted the data; Wrote the paper. Luxia Chen, Ruifang Han, Pen Hao, Liming Wang, Juan Gao, Xi Chen: Conceived and designed the experiments; Contributed reagents, materials, analysis tools or data. Xuan Li: Conceived and designed the experiments; Performed the experiments; Analyzed and interpreted the data.

## Data availability statement

Data included in article/supplementary material/referenced in article.

## Funding

This work was supported by the National Nature Science Foundation of China [32101101], the Science & Technology Foundation of Tianjin Eye Hospital [YKQN2006], Tianjin Science & Technology Foundation [16JCYBJC25800], Science and Technology Project of Tianjin Health Committee [TJWJ2021QN073], Tianjin Key Medical Discipline (Specialty) Construction Project and the Tianjin Health and Family Planning Communication Foundation [15KG120].

## Declaration of competing interest

The authors declare that they have no known competing financial interests or personal relationships that could have appeared to influence the work reported in this paper.
